# Celiac crisis in children in Serbia

**DOI:** 10.1186/s13052-016-0233-z

**Published:** 2016-03-01

**Authors:** Nedeljko Radlovic, Zoran Lekovic, Vladimir Radlovic, Dusica Simic, Biljana Vuletic, Sinisa Ducic, Zorica Stojsic

**Affiliations:** Faculty of Medicine, University of Belgrade, Dr Subotica 9, Belgrade, 11000 Serbia; University Children’s Hospital, Tirsova 10, Belgrade, 11000 Serbia; Academy of Medical Sciences of the Serbian Medical Society, Dzordza Vasingtona 19, Belgrade, 11000 Serbia; Pediatric Clinic, Clinical Centre, Zmaj Jovina 30, Kragujevac, 34000 Serbia; Institute of Pathology, Faculty of Medicine, University of Belgrade, Dr Subotica 1, Belgrade, 11000 Serbia

**Keywords:** Celiac crisis, Children, Developing country

## Abstract

**Background:**

To assess the prevalence and risk factors of celiac crisis (CC) in children with classical celiac disease (CD).

**Methods:**

This retrospective study comprised 367 children with classical CD diagnosed from 1994 to 2015. The diagnosis of CD was based on the revised ESPGHAN criteria and CC on acute worsening and rapid progression of chronic diarrhea and vomiting followed by severe dehydration, multiple metabolic derangements and a marked decrease of body weight.

**Results:**

Celiac crisis was confirmed in six (1.63 %) children, five in the first and one in the second year of life. In three patients CC was precipitated by rotavirus and in one by *Salmonella enteritidis* infection, while in the remaining two, except for a too long-standing disease and severe malnutrition, no additional causes of CC were found.

**Conclusion:**

Celiac crisis in Serbia is still-present in children exclusively below the second year of life as a spontaneous or intestinal infection precipitated complication of previously unrecognized CD.

## Background

Celiac crisis (CC) is an urgent and potentially fatal complication of celiac disease (CD) [1–4]. It is most often seen in early childhood, but may also occur later, including adult and elderly age group [1, 5–9]. It is clinically manifested by deterioration and rapid progression of serious digestive dysfunction followed by profuse watery diarrhea, severe dehydration and profound metabolic and nutritional abnormalities [1–5, 7, 10]. It is usually seen as a complication of previously unrecognized CD, and rarely as a consequence of non-compliance with the recommended gluten-free diet [3–5, 7].

We are presenting our experience related to the frequency of CC in children with classical CD in Serbia and the risk factors for its development. Additionally, we are reporting the therapy of this serious complication of CD.

## Methods

The study is retrospective and includes 367 children (240 female; with age range of 7 months to 15.25 years, mean 1.69 ± 1.48 years) with classical CD confirmed in two of the five reference centers in Serbia during the period from January 1994 to December 2015. The study protocol was approved by the local ethics committee. The classical type of CD implied chronic diarrhea, poor appetite and undernutrition [1, 2]. Most patients, (296, 80.65 %) were below the age of 2 years, of whom 129 were diagnosed in the first and 167 in the second year of life. In 346 patients the diagnosis of CD was based on the revised criteria of the European Society for Pediatric Gastroenterology, Hepatology and Nutrition (ESPGHAN) from 1989 [11], i.e. on the characteristic histopathological findings of small bowel mucosa and clinical recovery of patients on gluten-free diet, as well as on the confirmed clinical and/or histopathological relapse of the disease during gluten challenge at the age of 5–7 years in those diagnosed before completed second year of life. In 21 of them (13 female, age range of 10 months to 4.50 years, mean 1.82 ± 0.99 years) the diagnosis was based on the new ESPGHAN guidelines published in 2012 according to which there is no need for small intestinal biopsy in patients with signs or symptoms corresponding to CD, with IgA anti-tissue transglutaminase (anti-TTG) titers levels 10 times above the upper limit of normal, positive anti-endomysial antibody, presence of HLA-DQ2 and/or HLA-DQ8 and full clinical recovery on gluten-free diet [12]. All of them were diagnosed between 2012 and 2015.

Celiac crisis diagnosis was based on acute worsening and rapid progression of chronic diarrhea followed by severe dehydration, metabolic acidosis, hypotension, renal dysfunction, abdominal distension, hypoproteinemic edema and a marked decrease of body weight [1–3, 10].

Beside the history of the onset and course of the disease, a complete physical examination and standard laboratory investigations, all patients with CC underwent stool examination for bacterial infection, ova and parasites, latex agglutination test on rotavirus and adenovirus, duodenal fluid for *Giardia lamblia* and five serum testing for anti-TTG antibodies. In all six patients HLA genotyping was done as described in Stankovic et al. [13].

Initial therapy comprised of intravenous rehydration, correction of acidosis and hypoalbuminemia, interruption of oral feeding for the first 8–12 h, and in three cases with enormous abdominal distention a 1-day placement of a rubber tube in the rectum. To achieve full recovery of the patients, in addition to the strict gluten-free diet, supportive parenteral nutrition for 10 to 15-days and lactose-free diet for 2 to 3-weeks were necessary. Due to persistent secretory diarrhea and perpetual anorexia requiring a continual intravenous fluid substitution, in two cases, both without clinical and laboratory indications of infection, a 5-day oral prednisone therapy in the dose of 2 mg/kg daily was also necessary.

After 2 weeks of treatment, small intestinal biopsy was performed and histological findings were classified according to the modified Marsh criteria [14].

## Results

Celiac crisis was detected in six (1.63 %) children (4 female; aged 8 to 15 months, mean 10.50 ± 2.59 months), all at the time of initial presentation of classical CD. Five patients were in the first and one in the second year of life, thus the prevalence of CC for this age group was 2.03 %, i.e. 3.88 % for the first and 0.60 % for the second one. Year of hospitalization and basic clinical data of patients with CC are presented on Table 1. Laboratory findings on admission showed mild metabolic acidosis (pH capillary blood 7.21–7.26; 7.23 ± 0.02), low serum levels of albumin (18–24 (21.50 ± 2.81 g/L), sodium ((123–129; 126 ± 2.90 mmol/L), potassium (2.1–2.9; 2.45 ± 0.339 mmol/L), calcium (1.02–1.98; 1.59 ± 0.42 mmol/L), magnesium (0.61–0.68; 0.65 ± 0.026 mmol/L) and phosphorus (0.62–0.79 (0.69 ± 0.07 mmol/L), high serum creatinine (96–133; 107.33 ± 13.53 μmol/L) and anemia (Hb 71–98; 85.17 ± 9.67 g/L).

**Table 1:** Basic data for children with CC

Case	Year of hospitalization	Duration of breast feeding (mths.)	Age at gluten introduction (mths.)	Age at onset of CD symptoms (mths.)	Age at diagnosis (mths.)	Deficit of BW on admission (%)	Edema
1	1999	2	3.5	12.5	15	22.5	+
2	2004	1.5	3	8	10	32	+
3	2004	0.3	3	6	8	33	+
4	2005	1	3.5	7.5	9	28	+
5	2009	0.5	3	7.5	9	28	+
6	2010	0.25	4	11	12	24	+

*BW* body weight

In four patients CC was probably precipitated by intestinal infection caused by rotavirus in three and by *Salmonella enteritidis* in one, while in the remaining two, except for a too long-standing disease and severe malnutrition, no additional causes of CC were found.

**Fig. 1:**
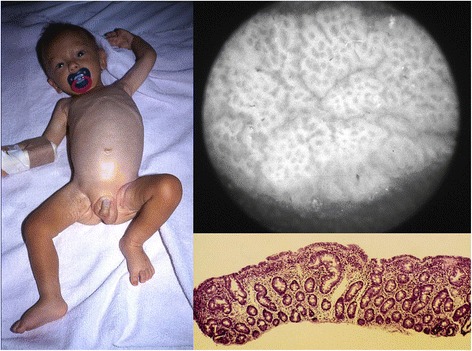
Male patient in initial phase of recovery and it’s stereomicroscopic and histological appearance of the small intestinal mucosa

In all six cases the stereomicroscopic and histological examination of the small intestinal mucosa showed a total villous atrophy (Marsh IIIc) (Fig. 1) and were homozigous carriers of HLA-DQ2.5 haplotype characterised by HLA-DRB1*03, HLA-DQA1*05 and HLA-DQB1*02 alleles present on a single (in cis configuration). Additionally, all five tested were positive to IgA anti-TTG antibodies (58.6 to 88 U/ml).

## Discussion

Owing to advancement in knowledge and diagnostics of CD, CC is rare today, even in the developing countries [3, 5]. However, there is no any publication either in pediatric or adult medicine precisely tackling the issue of its current incidence [7]. Besides, there are no papers, review or original, which systematically consider the basis of the occurrence and possibilities of CC treatment at this point of time.

Our analysis originating from Serbia, which is a developing country, indicates that CC in children is rare today, and that it is seen only by the end of the first and the beginning of the second year of life as a spontaneous or precipitated complication of unrecognized on time and neglected CD. In our three patients the precipitated factor of CC was probably rotavirus gastroenteritis and in one *Salmonella enteritidis*, while in the remaining two, except for a too long-standing disease and severe malnutrition, no additional causes of CC were determined. The significance of intestinal infection as one of the precipitating factors of CC in patients with active CD and marked malnutrition is well known and is reflected, not only by a rapid deterioration of already disordered digestive functions, but also by severe anorexia and fever induced hypermetabolic condition. In three of our patients this is particularly marked in intercurrent rotavirus gastroenteritis, where both autoimmune and infective mechanisms contribute to the proximal small bowel segment mucosa damage, i.e. in the segment that plays the central role in food digestion and absorption [15]. It is the fact that intestinal infections are pointed out as a precipitating factor of CC, however, to our knowledge the available literature does not mention rotavirus gastroenteritis specifically. Additionally, all six children were homozigous carriers of HLA-DQ2.5 haplotype as a high-risk for early expression of CD and possibility of CC as its complication at this age [16, 17].

The first therapeutic procedures were aimed at vital care and stabilization of the patients’ condition, while for their definite recovery, beside the standard measures, in two patients a short-term prednisone therapy was also necessary. According to other authors’ experience, although risky, steroids used to treat resistant forms of CC have proved successful [5, 6, 18, 19].

The explanation for the fact that CC in our two institutions registered in the middle of the observed period, i.e. from 1999 to 2010, probably lies in the fall of economic standard in the country in this context and the level of healthcare. However, in the last 5 years we did not have any patient with CC, which can be explained by a more stable situation in the country in general, and in this context, and improvement of CD diagnosis.

## Conclusion

Celiac crisis in Serbia is still-present in children and occurs exclusively by the end of the first or the beginning of the second year of life as a spontaneous or intestinal infection precipitated complication of classical CD unrecognized on time.

Written informed consent was obtained from the patient’s legal guardian(s) for publication of this case report and any accompanying images. A copy of the written consent is available for review by the Editor-in-Chief of this journal.
